# Supplementary material for: Volatile organic compound patterns predict fungal trophic mode and lifestyle
Source: Commun Biol. 2021 Jun 3;4:673. doi: 10.1038/s42003-021-02198-8 (PMC8175423; doi:10.1038/s42003-021-02198-8)
Supplement: Supplementary file 2 — Supplementary information [file 42003_2021_2198_MOESM2_ESM.pdf]

Volatile organic compound patterns predict fungal trophic mode and lifestyle

Yuan Guo, Werner Jud, Fabian Weikl, Andrea Ghirardo, Robert R. Junker, Andrea Polle, J. Philipp Benz, Karin Pritsch, Jörg-Peter Schnitzler, Maaria Rosenkranz

Supplementary Information

Supplementary Figure S1

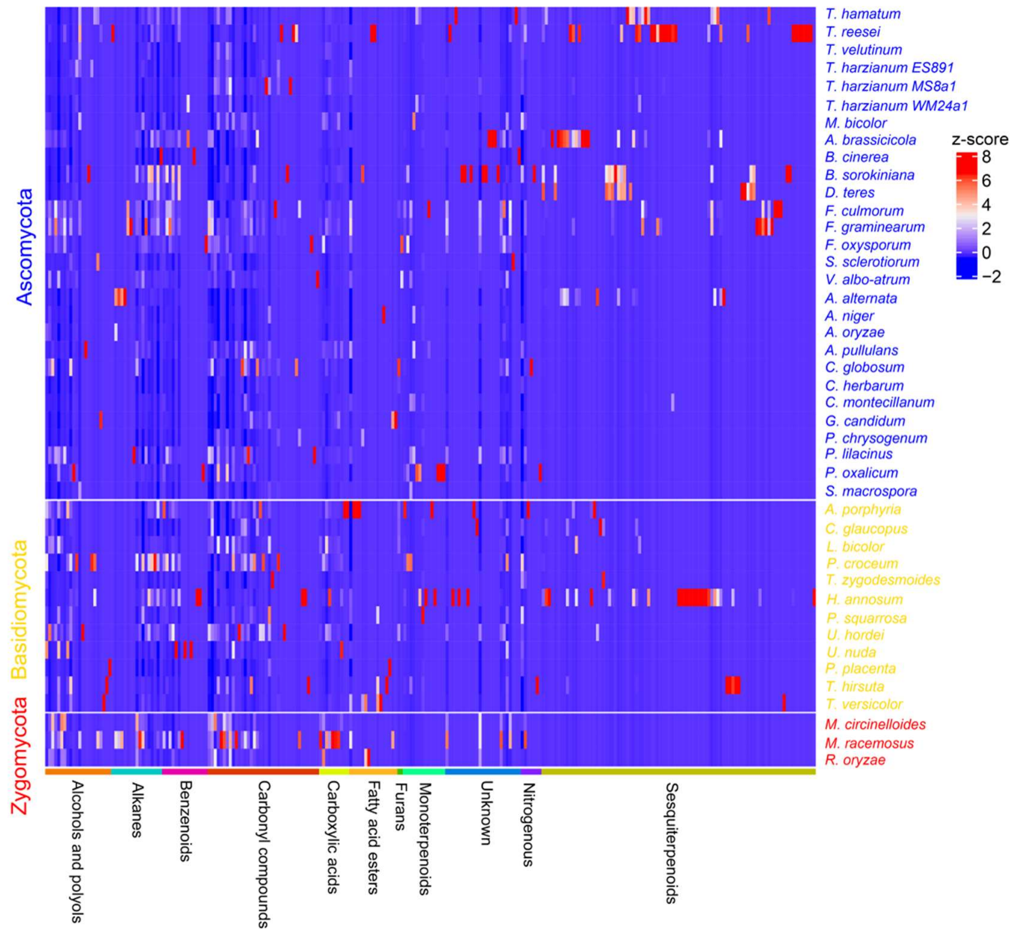

Fig. S1. Heatmap of fungal volatile organic compounds (fVOC) emission profiles from the examined 43 fungal species. The emission intensity of fVOCs grouped in structural classes is shown for each fungal species. The emission rates (ncps cm^{-2} s^{-1} and pmol cm^{-2} h^{-1}) based on PTR-MS and GC-MS data, respectively, are color coded: red indicates high and blue indicates low emission.

Supplementary Figure S2

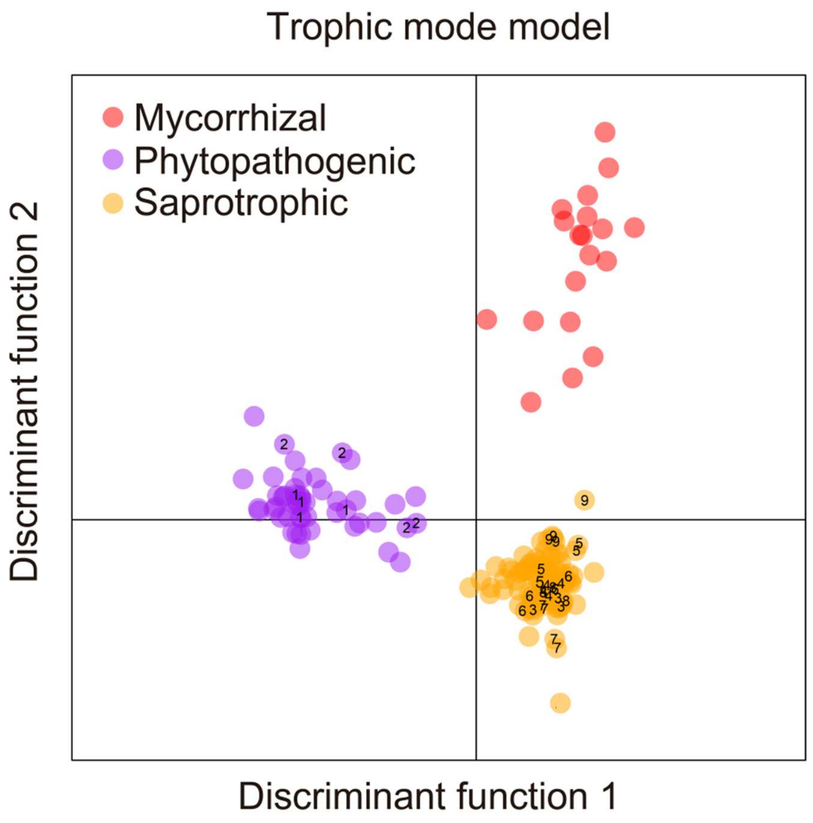

Fig. S2. Functional characterization of fungi using the complete fungal volatile organic compound (fVOC) profiles. The discriminant analysis of principal components (DAPC) model comprise trophic mode when an alternative grouping of the fungal species to different guilds is used. The numbers 1-7 denote the fungal species for which the trophic mode was changed to the alternative group (i.e. *Alternaria alternata* (#1) and *Aspergillus niger* (#2) were moved from saprophytes to phytopathogens, *Fusarium oxysporum* (#3) from phytopathogens to saprophytes and *Trichoderma* spp. (#4-7) from mycoparasites to saprophytes; for details of the alternative guild and related references please see Supplementary Table S1[1]). The results show scatter plots with dots denoting individual fungus. Detailed parametric configurations of DAPC models are provided in the materials and methods.

Supplementary Figure S3

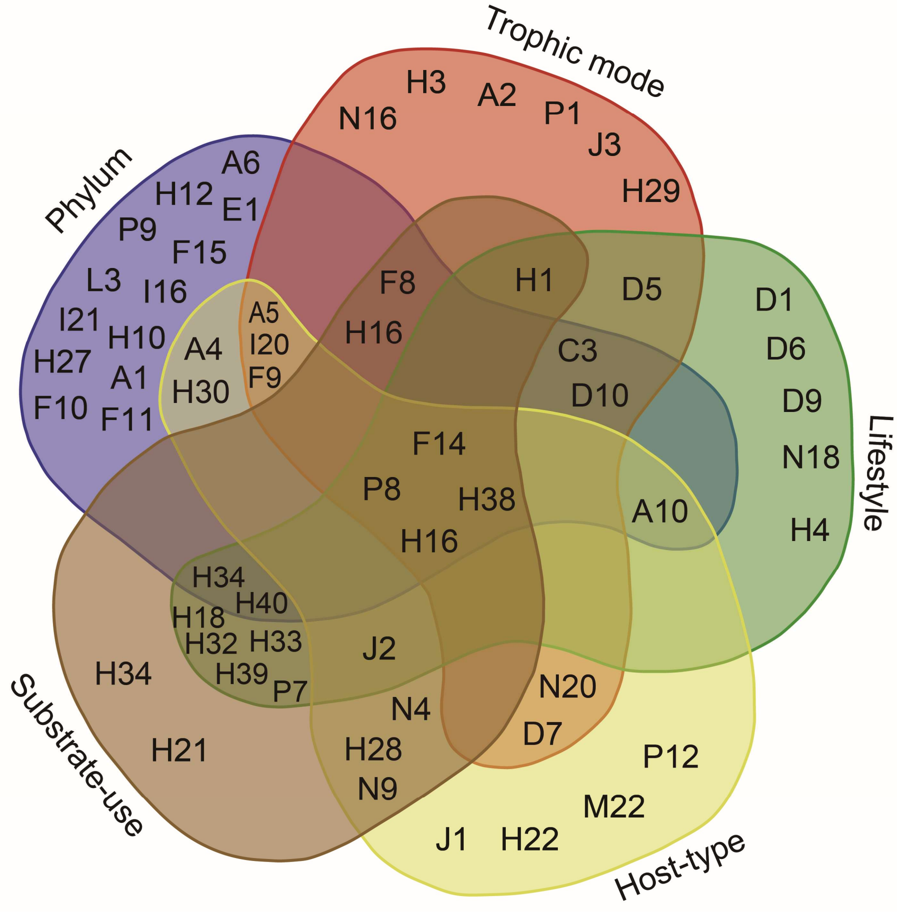

Fig S3. The Venn plot indicates the number of unique and shared fungal volatile organic compounds (fVOCs) between fungi grouped according to phylum, trophic mode, lifestyle, substrate-use and host-type. The letter-number combinations refer to the compounds listed in Supplementary Tables S2 and S3[1].

Supplementary Figure S4

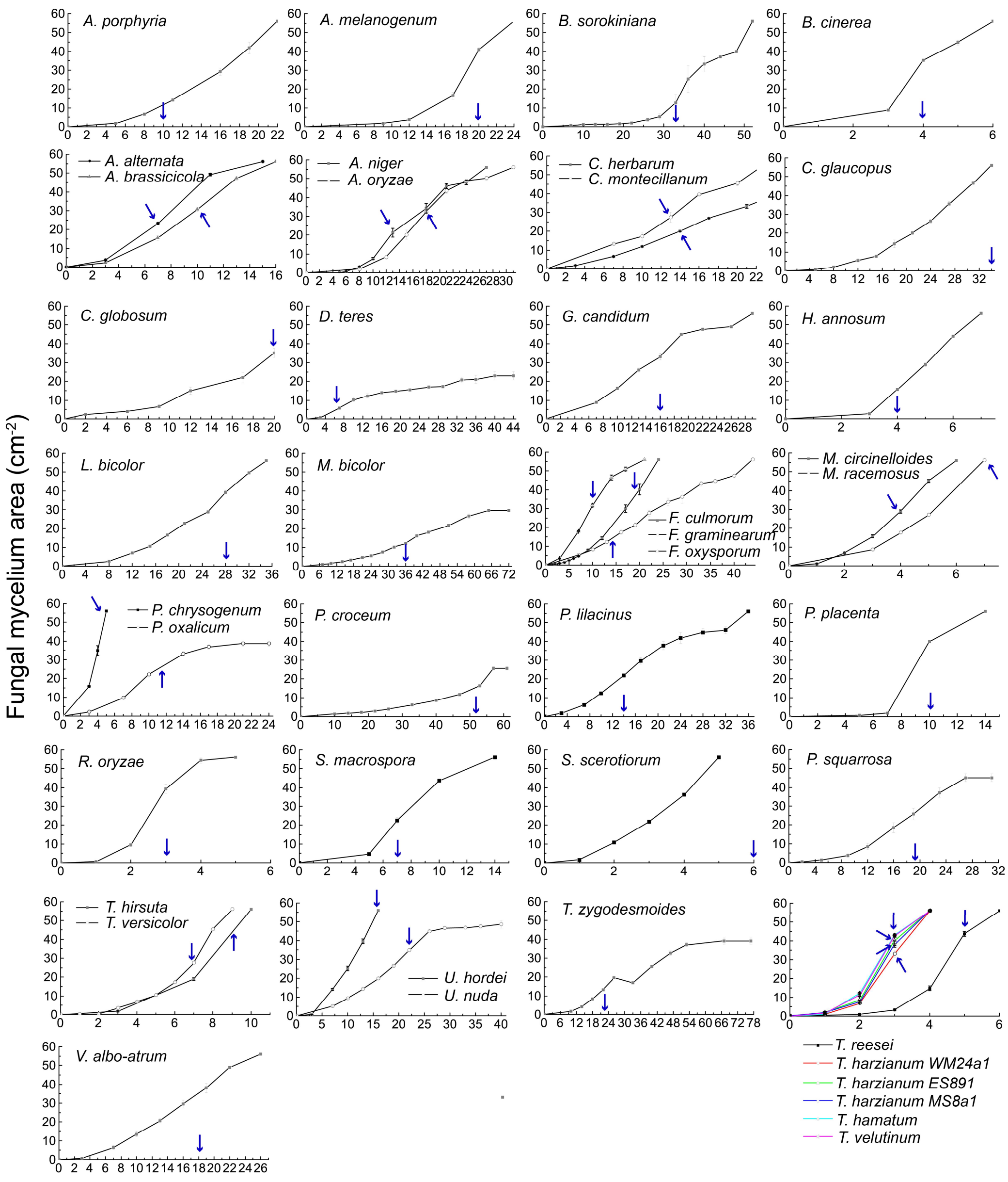

Fig. S4. Growth curves of the 43 investigated fungi. The time points for emission measurements were chosen to lie in the exponential growth stage of each fungus and were determined in preceding experiments from the maximum of the first derivative of the growth curve. Blue arrows indicate the time points for the start of fungal volatile organic compound (fVOC) measurements. Data are shown as means ± SE (n = 5 biologically independent samples).

References:

1 Supplementary Data: Supplementary Tables S1-S6. DOI:10.17605/OSF.IO/BVA2Q https://osf.io/bva2q/
